# Metabolic syndrome among type 2 diabetic patients in Ethiopia: a cross-sectional study

**DOI:** 10.1186/s12872-018-0880-7

**Published:** 2018-07-17

**Authors:** Mequanent Kassa Birarra, Dessalegn Asmelashe Gelayee

**Affiliations:** 1 Department of Clinical Pharmacy, School of Pharmacy,College of Medicine and Health Sciences, University of Gondar, Lideta Street, P.o.box: 196, Gondar, Ethiopia; 2 Department of Pharmacology, School of Pharmacy,College of Medicine and Health Sciences, University of Gondar, Gondar, Ethiopia

**Keywords:** Metabolic syndrome, Type 2 diabetes mellitus, University of Gondar, Ethiopia

## Abstract

**Background:**

Metabolic syndrome (MetS) increases risk of cardiovascular diseases (CVD), premature death as well as cost related to health care.This study was aimed at investigating the prevalence of MetS and its determinant factors among type2 diabetes mellitus (T2DM) patients attending a specialized hospital.

**Methods:**

A cross-sectional study was conducted on a total of 256 T2DM patients from the first march to 30th May 2017 at university of gondar comprehensive specialized hospital (UGCSH). Data was collected based on STROBE (strengthening the reporting of observational studies in epidemiology) statement. Bivariable and multivariable logistic regression analysis were run to identify predictors of MetS from the independent variables and significance test was set at *P* <  0.05.

**Results:**

The prevalence of MetS in this study was 70.3, 57 & 45.3% and it is more common in females (66.1, 83.3 & 70.7%) by using national cholesterol education program adult treatment panel III (NCEP-ATP III), International diabetic federation (IDF) and world health organization (WHO) criteria respectively. The most prevalent components of MetS were low level of high density lipoprotein (HDL) and triglyceride(TG). By usingIDF criteria, female gender was significantly associated with MetS (AOR = 0.2 at 95%CI: 0.1, 0.6 *P* = 0.00). Where as by NCEP-ATP IIIcriteria, age between 51 and 64 years old (AOR = 2.4 95% CI: 1.0,5.8, *P* = 0.04), self employment (AOR = 2.7 95% CI:1.1, 6.5, *P* = 0.03), and completetion of secondary school and above (AOR = 3.2, 95% CI:1.6,6.7, *P* = 0.001) were predictors for the development of MetS. In the WHO criteria, being single in marital status was significantly associated with MetS (AOR = 17 at 95%CI: 1.8, 166, *P* = 0.000).

**Conclusions:**

This study demonstrates that Metabolic syndrome is a major health concern for diabetic patients in Ethiopia and they are at increased risk of developing complications such as cardiovascular diseases and premature mortality. The predictors female gender, age between 51 and 64 years old, urban area residence, and being single are modifiable.Thus,health authorities shall provide targeted interventions such as life style modifications to these most at risk sub-populations of diabetic patients.

## Background

The burden of non-communicable disease in the developing countries is increasing, and leading to high mortality rates [1]. Nowadays T2DM is pandemic and there are no signs of reduction in the incidence rates [2]. Forexamle, according to international diabetes federation report indicates that more than 415 million of people worldwide adults have diabetes. By 2040 this will rise to 642 million. In Africa, 441 million people live with diabetes which is likely to increase by 926 million in 2040 [3]. Diabetic population are at increased risk of mortality and morbidity primarily due to cardiovascular diseases [4]. The relative risks are 1 to 3 in men and from 2 to 5 in women [5]. Metabolic syndrome would have its own contribution in these outcomes of DM. Metabolic syndromeis highly prevalent in T2DM patients [6–8]. However, several studies have reported lower prevalence of MetS [9, 10] and this is largely due to differences in characteristics of the studied population such as residence, type of disease and comorbidities, etc.

Metabolic syndrome can be defined as a cluster of interconnected cardio-metabolic dysfunctions which is characterized by the increase in fasting blood sugar (FBS), abdominal circumference (AC), arterial pressure (AP), triglycerides (TG), and reduction in high-density lipoprotein cholesterol (HDL) [11]. This syndrome has different set of criterias to measure it. Those are National Cholesterol Education Program Adult Treatment Panel III (NCEP-ATP III) [12], WHO criteria’s [13] and IDF [14]. The NCEP-ATP III definition uses the presence of 3 or more parameters as a cutoff to define MetS and the WHO as well as IDF definitions require the presence of at least two parameters.

The syndrome can directly contributes to the development of CVD and the appearance of T2DM in non-diabetic patients. Additionally, it increases the risk of premature death, renal disease, mental disorders and cancer. Thus MetS represents a serious public health problem [15–17].

Metabolic syndrome is not also with out cost implications. For instance, Boudreau et al. found that costs for subjects with diabetes plus weight risk, dyslipidemia, and hypertension were almost double the costs for subjects with prediabetes plus similar risk factors ($8067 vs. $4638) [18].

Globally, 20–25% of the adult population has MetS and they are twice as likely to die from it; and they are three times more likely to have a heart attack or stroke compared with people without the syndrome [14, 19]. However, the prevalence of MetS in type 2 diabetes in sub-Saharan Africans according to two sets of diagnostic criteria was 71.7% according to the IDF criteria and 60.4% using NCEP-ATP III criteria [20]. In Ethiopia the prevalence of MetS was range from (26–70%) using NCEP-ATP III criteria [21–23].

Nowadays, MetS has become a significant public health problem. Therefore, there is a need for investigation in this area [24]. Taking into consideration, diabetic patients who had MetS also they have cardiovascular risk factors, therefore the diagnosis of MetS in those patients is very important for detection, prevention, and treatment of the underlying risk factors and for the reduction of the cardiovascular disease burden in the general population [25, 26].

While limited studies of MetS among diabetic patients in Ethiopia acknowledge its burden, they followed a single criteria(NCEP-ATP III) to define MetS and using a signle criteria may either under or over estimate the problem. Thus denying or providing interventions to minimize the risks of MetS complications would be irrational since a given patient may be categorized as having MetS in one set of definition but not in the others. In this regard, the present study employed three commonly used criteria to define MetS so that it would be easy to acknowledge the importance of having a unified MetS criterion to make appropriate clinical decision in the context of Ethiopia.Therefore, this study was aimed at inevestigating the prevalence of MetS and its determinant factors among T2DM patients attending a comphrensivespecialized hospital.

## Methods

### Study Area & Period

The study was conducted from March to May 2017 at UGCSH, Northwest Ethiopia. The hospital is currently serving more than 5 million people in the surrounding area andit is located in Gondar town, 750 km Northwest of the capital city. It has more than 400 beds and fourteen different units that provide medical services to nearly 250,000 out-patients each year. More than 5 thousand diabetic patients attend the diabetic follow up clinic.

### Study design and population

An institutional based cross sectional study design was followed.The source populations were all patients attending the facility on out-patient basis at UGCSH.Whereas, all adult T2DM patients attending the facility on out-patient basis during the study period and volunteered to take part in the study were the study population. Those patients whose age ≥ 20 years old and diagnosed as T2DM undergoing treatment with the facility were included in the study. Whereas, pregnant women, excessive alcohol or other drug abuse, having current psychiatric treatment and incomplete patient’s data were excluded from the study.

### Sample size and sampling procedure

The sample size was calculated based on single population proportion formula [27]. By using the following assumption: (1.96)^2^ were used for $Z\frac{\alpha }{2}$ and the proportion (P) of MetS in these groups was 0.5. With 95% confidence interval (CI) and marginal error (d) of 5%.

$$ n=\frac{Z_{\frac{\alpha }{2}}{}^2P\left(1-P\right)}{d^2} $$

Based on the above formula, assumptions, correction formula and 5% of contingency the sample size(n) was calculated to be 256. Study participants were selected using systematic random sampling technique. Then, every third patient arrived at the clinic was selected for the study.

### Data collection procedure

Data of socio demographic and economic (age, sex, monthly income, life style, family history of diabetes and other diseases/disorders) of the study participants were collected by using standardized interview questioner.Whereas, data of HDL, fasting plasma glucose (FPG), and TG were recorded from patient files and chart.The components of MetS was identified and determined according to NCEP-ATP III, IDF and WHO definitions. Anthropometric data of the study participants (weight, height and waist circumference) was obtained by two data collector nurses who are working at UGCSH diabetic clinics. Weight was obtained from patients using weight balance while they were visiting the clinics during their follow-up. The average follow-up interval was 2–3 months. Height of the patients were assessed using meter and also data collectors were instruct participants to stand upright, motionless,and touching their thighs with their palms. Based on height and weight body mass index was calculated. Waist circumference (WC) was measured midway between the inferior angle of the ribs and the supra-iliac crest by using Meter [28]. After 10 min of arrival of the study participants at UGCSH diabetic clinic, Blood pressure (BP) was measured using a standard adult arm cuff of mercury type sphygmomanometer by the recruited nurses as data collectors who working in theclinic. Inorder to assure the reliability of BP measurement data collectors were taken two readings with 1 minute interval and the average of the two readings was recorded as the final BP of the patient. However, a third measurement was taken if the difference between the two readings was greater than 5 mmHg and the average of the 3 BP readings was recorded as the final BP of the patient [29].

### Data quality assurance

In order to control the quality of data, pre-test was done in the data abstraction format before the main data collection on a sample equivalent to 13 (5%) of the total sample size in randomly selected patients. The pretested papers were not included in the study and appropriate adjustment was done on the data abstraction format. In addition to this the principal investigator had supervised the data collectors during data collection. Thenthe collected data were checked for completeness and consistency on daily basis.

### Data analysis and interpretation

The collected data were entered into Epi Info version 7 and exported to statistical package for the social sciences (SPSS) version 20 for statistical analysis. The results presented using tables and figures. Frequency distribution was calculated. The prevalence of patients with MetS was calculated, dividing the number of patients with MetS by the total number of study participants. To identify factors independently associated with the occurrence of MetS Bivariable and multivariable logistic regression analysis was run. The results of Bivariable and multivariable analysis were reported as crude and adjusted odds ratio at 95% confidence intervals (95% CI) and *P*-value ≤0.05 was considered as statistical significance.

### Operational definitions

#### NCEP-ATP III criteria

Study participants were classified as having MetS if they had three or more of the following risk factors: waist circumference (> 102 cm for men and > 88 cm for women), high plasma triglycerides (≥ 150 mg/dl), low HDL cholesterol (< 40 mg/dl for men and < 50 mg/dl for women), blood pressure (≥ 130/85 mmHg) and fasting plasma glucose (≥110 mg/dl) [12].

#### WHO criteria

Study participants were classified as having MetS as along with DM if they had any two of the following components: Obesity: BMI (> 30 kg/m2), high serum triglycerides, (≥150 mg/dl), low serum high density lipoprotein cholesterol (< 35 mg/dl for men and < 39 mg/dl for women) and having hypertension (≥140/90 mmHg [13].

#### IDFcriteria

Study participants were classified as having MetS as along with central obesity if they had any two of the following components:Raised TG levels ≥150 mg/dl (1.7 mmol/l), or specific treatment for this lipid abnormality, reduced HDL-cholesterol < 40 mg/dl (1.03 mmol/l) in males and < 50 mg/dl (1.29 mmol/l) in females, or specific treatment for this lipid abnormality, raised blood pressure: systolic BP ≥130 or diastolic BP ≥85 mmHg or treatment of previously diagnosed hypertension, raised fasting blood glucose ≥100 mg/dl (≥5.6 mmol/l) or previously diagnosed diabetes and waist circumference (> 94 cmfor men and > 88 cm for women [14].

### Body mass index (BMI)

Was defined as the ratio between weight (kg) and the square of the height (m) and used to categorize BMI-measured weight status: patients with (BMI ≤ 18.5) statedas under weight, patients with (BMI 18.5–24.9) consider as normal,however, patients with (BMI 25.0–29.9) is overweight and obese if BMI is ≥30 [22].

## Results

### Socio-demographic characteristics

A total of 256 study participants were included in the study of which more than half of them were females 143 (55.9%). The highest number of study participants were in the age group (51–64) years old. More than three fourth were 207 (80.9%) lives in urban area and 93 (36.3%) of them were complete their secondary school and above. In addition, more than two third of 219 (85%) were married.

The total number of unemployed study participants were 131 (51.2%) and majority of them 116 (45.3%) had < 600 Ethiopian birr monthly income. The highest number (81.6%) of them used palm oil for food preparation. In addition to this, majority of study participants 136 (53.1%) were not involved in work vigarious intensity of activity and the highest number 173 (67.6%) of them were not did regular physical exercise. One hundred sixty seven (62.2%) of study participants have no family history of chronic diseases. Around half of the study subjects 136 (53.1%) diagnosed DM between 1 and 5 years duration and all of them were under medication. Most of them 132 (51.5%) were also undertaking combination treatment. Details are presented in Table 1**.**

**Table 1:** Socio demographic characteristics of the study participants at UGCSH, June 2017

Variables		Frequency	Percentage (%)
Sex	Male	113	44.1
	Female	143	55.9
Age (years)	≤ 30	27	10.5
	31–40	15	5.9
	41–50	56	21.9
	51–64	92	35.9
	≥ 65	66	25.8
Residency	Urban	207	80.9
	Rural	49	19.1
Educational status	Unable to read and write	76	29.7
	Primary school	87	34
	Secondary school and above	93	36.3
Marital status	Single	18	7
	Married	219	85.5
	Divorced	7	2.7
	Widowed	12	4.7
Occupation	Government	78	30.5
	Unemployed	131	51.2
	Self employed	47	18.4
Monthly income(ETB)	< 600 birr	116	45.3
	601-1500birr	68	26.6
	> 1500 birr	72	28.1
Types of Oil used	Palm Oil	209	81.9
	Cereal Oil	46	18.9
Work involving vigorous intensity of activity	Yes	120	46.9
	No	136	53.1
Regular physical exercise	Yes	83	32.4
	No	173	67.6
Family history of chronic disease	Yes	89	34.8
	No	167	65.2
Duration since diabetic mellitus diagnosed	< 1 year	22	8.6
	1–5 years	136	53.1
	> 6 years	98	38.3
Anti diabetic medication started	Yes	256	100
	No	0	0
Duration since anti Diabetic started	< 1 year	24	9.4
	1–5 years	135	52.7
	> 6 years	97	37.9
Total number of medications taken	1	113	44.1
	2	85	33.2
	3	40	15.6
	4	7	2.7

### Prevalence of metabolic syndrome with each criteria

**Fig. 1:**
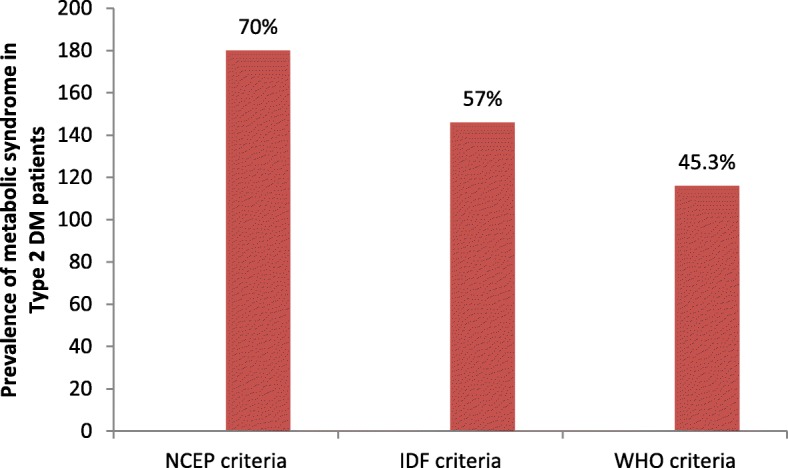
Metabolic syndrome in different criteria at UGCSH, June 2017

The prevalence of MetS in this study was 180 (70.3%), 146 (57%) and 116 (43.3%) using NCEP(ATPIII), IDF and WHO criteria respectively (Fig. 1).

### Frequency of metabolic syndrome components by sex

The frequency of MetS components in this study based on NCEP-ATP III criteria were 53.5, 68.8 and 67.2% for abdominal obesity, elevated triglyceride and reduced HDL respectively. Whereas, using the IDF criteria the prevalence was 61.7, 67.6 and 66.8% for abdominal obesity, elevated triglyceride and reduced HDL respectively. Details are presented in Table 2**.**

**Table 2:** Frequency of metabolic syndrome components among T2DM patients with sex, at UGCSH, June 2017

Metabolic components	Criteria for MetS	N (%)	Male	Female	*P* value
Abnormal obesity	IDF	42 (16.4)	6 (5.3%)	36 (25.2%)	< 0.0001
	WHO	42 (16.4)	7 (6.2%)	35 (24.5%)	< 0.0001
Abnormal fasting	IDF	239 (93.4%)	104 (92%)	135 (94.4%)	0.452
	NCEP	236 (92.2%)	104 (92%)	132 (92.3%)	0.936
Abnormal HDL	IDF	171 (66.8%)	58 (51.3%)	113 (79%)	< 0.0001
	WHO	86 (33.6%)	22 (19.5%)	64 (44.8%)	< 0.0001
	NCEP	172 (67.2%)	58 (51.3%)	114 (79.7%)	< 0.0001
Abnormal TG	IDF	173 (67.6%)	65 (57.5%)	108 (75.5%)	< 0.002
	WHO	172 (67.2%)	64 (56.6%)	108 (75.5%)	< 0.002
	NCEP	176 (68.8%)	66 (58.4%)	110 (76.9%)	< 0.002
Abnormal WC	IDF	158 (61.7%)	45 (39.8%)	113 (79.0%)	< 0.0001
	NCEP	137 (53.5%)	24 (21.2%)	113 (79.0%)	< 0.0001
Abnormal BP	IDF	110 (43.0%)	46 (40.7%)	64 (44.8%)	0.516
	WHO	61 (23.8%)	26 (23%)	35 (24.5%)	0.784
	NCEP	111 (43.4%)	47 (41.6%)	64 (44.8%)	0.612

### Factors associated with metabolic syndrome

In order to control confounders effect multivariable logistic regression analysis was run to analyze variables which were significantly associated to different components of MetS using different criteria in bivariable logistic analysis. These variables were sex, age, educational status, residency, duration since DM diagnosed, monthly income, family history of chronic disease and marital status. The analysis showed that, sex was significantly associated with MetS by using IDF criteria. Based on this, female patients were (AOR = 0.2 at 95%CI: 0.1, 0.6, *P* = 0.00) significantly associated with MetS compared to men using IDF criteria. Details are presented in Table 3.

**Table 3:** Bivariable and multivariable logistic regression analysis by using IDF criteria at UGCSH, June 2017

Variable		Mets		COR 95%CI	*P*-value	AOR 95% CI	*P*-value
		Yes	No				
Sex	Male	40 (27.4%)	105 (50.2%)	1.00	0.00	0.2 (0.1,0.6)	< 0.00*
	Female	106 (72.6%)	104 (49.8%)	0.2 (0.1,0.4)			
Age	< 30	0 (0%0	27 (100%)	1.00	–		
	31–40	2 (13.3%)	13 (86.7%)	0.0 (0.0,-)	0.9		
	41–50	9 (16.1%)	47 (83.9%)	0.4 (0.9,2.2)	0.3		
	51–64	19 (20.7%)	73 (79.3%)	0.5 (0.2,1.4)	0.2		
	> 65	17 (25.8%)	49 (74.2%)	0.8 (0.4,1.6)	0.5		
Residency	Urban	40 (19.3%)	167 (80.7%)	0.7 (0.3,1.7)	0.41		
	Rural	7 (14.3%)	42 (85.7%)	1.00	–		
Educational status	Unable to read and write	22 (46.8%)	54 (25.8%)	0.4 (0.2,0.9)	0.03	–	–
	Primary school	13 (27.7%)	74 (35.4%)	1.00	–	1.3 (0.7,3.6)	0.2
	Secondary school and above	12 (25.5%)	81 (38.8%)	0.4 (0.2,0.8)	0.01	1.4 (0.5,3.3)	0.4
Marital status	Single	4 (8.5%)	14 (6.7%)	0.4 (0.1,1.3)	0.1		
	Married	35 (74.5%)	184 (88%)	2.7 (0.4,18.1)	0.3		
	Divorced	4 (8.5%)	3 (1.4%0	1.00	–		
	Widowed	4 (8.5%)	8 (3.8%)	0.57 (0.1,2.9)	0.5		
Occupation	Government	13 (27.7%)	65 (31.1%)	1.00	–		
	Unemployed	29 (61.7%)	102 (48.8%)	0.4 (0.2,1.2)	0.09		
	Self employed	5 (10.6%)	42 (20.1%)	0.7 (0.3,1.4)	0.3		
Monthly income(ETB)	< 600 birr	21 (44.7%)	95 (45.5%)	1.00	–		
	601-1500 birr	13 (27.7%)	55 (26.3%)	1.0 (0.5,2.2)	0.9		
	> 1501 birr	13 (27.7%)	59 (28.2%)	1.1 (0.5,2.5)	0.8		
Types of Oil used	Palm Oil	38 (80.9%)	172 (82.3%)	1.1 (0.5,2.5)	0.8		
	Cereal Oil	9 (19.1%)	37 (17.7%)	1.00	–		
Work involving vigorous intensity of activity	Yes	21 (44.7%)	26 (55.3%)	1.00	–		
	No	99 (47.4%)	110 (52.6%)	0.9 (0.5,1.7)	0.7		
Regular physical exercise	Yes	13 (27.7%)	34 (72.3%)	1.00	–		
	No	70 (33.5%)	139 (66.5%)	0.8 (0.4,1.5)	0.4		
Family history of chronic disease	Yes	21 (44.7%)	26 (55.3%)	0.6 (0.3,1.1)	0.1		
	No	68 (32.5)	141 (67.5%)	1.00	–		
Duration since Diabetic mellitus diagnosed	< 1 Year	4 (8.5%)	18 (8.6%)	1.00	–		
	1-5 Years	25 (53.2%)	111 (53.1%)	0.9 (0.5,1.5)	0.9		
	> 6 Years	18 (38.3%)	80 (38.3%)	0.9 (0.3,3.1)	0.9		
Duration since anti Diabetic started	< 1 Year	5 (10.6%)	19 (9.1%)	1.00	–		
	1-5 Years	25 (53.2%)	110 (52.6%)	0.9 (0.4,1.8)	0.8		
	> 6 Years	17 (36.2%)	80 (38.3%)	1.1 (0.4,3.4)	0.8		
Total number of medications	1	94 (45.0%)	1.00	–	–		
	2	79 (37.8%)	0.8 (0.16,4.1)	0.8	–		
	3	28 (13.4%)	0.7 (0.1,3.5)	0.7	–		
	4	8 (3.8%)	1.9 (0.3,9.9)	1.9	–		

*P < 0.05

Using NCEP-ATPIII criteria, female sex was (AOR = 0.2 at 95%CI: 0.1, 0.6, *P* = 0.00) significantly associated with MetS compared to male sex. Similarly, patients whose age is between 51 and 64 years old were about two (AOR = 2.4 95% CI: 1.0, 5.8, *P* = 0.04) times more likely to haveMetS compared to those patients whose age is < 30 years old. Likewise,self employed participants were about three (AOR = 2.7 95% CI: 1.1, 6.5, *P* = 0.03) times more likely to develop MetS compared to those unemployed. Patients who completed secondary school and above were about three (AOR = 3.2, 95% CI: 1.6, 6.7, *P* = 0.001) times more likely to develop MetS compared to those unable to read and write. In addition, patients whose DM diagnosis duration was less than 1 year were about three (AOR = 2.7 95% CI = 1.1, 7.1, *P* = 0.04) times more likely to develop MetS compared to those with DM diagnosis duration 1–5 years. Details are presented in Table 4.

**Table 4:** Bivariable and multivariable logistic regression analysis using NCEP-ATPIII criteria at UGCSH, June 2017

Variable		Mets		COR 95%CI	*P*-value	AOR 95%CI	*P*-value
		Yes	No				
Sex	Male	61 (33.9%)	52 (68.4%)	1.00	–		
	Female	119 (66.1%)	24 (31.6%)	0.23 (0.1,0.4)	0.00	0.2 (0.1,0.5)	< 0.00*
Age	< 30	14 (7.8%)	13 (17.1%)	1.00	–		
	31–40	9 (5%)	6 (7.9%)	0.2 (0.1,0.6)	0.04	3.8 (1.4,10.9)	< 0.01*
	41–50	37 (20.6%)	19 (25.0%)	0.3 (0.1,1.1)	0.08	4.5 (1.2,16.4)	< 0.02*
	51–64	66 (36.7%)	26 (34.2%)	0.4 (0.2,0.1)	0.05	2.4 (1.0,5.8)	< 0.04*
	> 65	54 (30.0%)	12 (15.8%)	0.5 (0.3,1.22)	0.14	1.9 (0.9,4.4)	0.1
Residency	Urban	152 (84.4%)	55 (72.4%)	0.5 (0.3,0.9)	0.02	2.8 (1.4,5.6)	< 0.004*
	Rural	28 (15.6%)	21 (27.6%)	1.00	–	–	–
Educational status	Unable to read and write	59 (32.8%)	17 (22.4%)	0.4 (0.2,0.8)	0.008	1.2 (0.6,2.5)	0.6
	Primary school	67 (37.2%)	20 (26.3%)	1.00	–		
	Secondary school & above	54 (30,0%)	39 (51.3%)	0.1 (0.5,2.0)	0.92	3.2 (1.6,6.7)	< 0.001*
Marital status	Single	10 (5.6%)	8 (10.5%)	0.00 (0.0,-)	0.9		
	Married	151 (83.9%)	68 (89.5%)	1.0 (0.0,-)	1.0		
	Divorced	7 (3.9%)	0 (0.0%)	1.00	–		
	Widowed	12 (6.7%)	0. (0.0%)	0.00 (0.0,-)	0.9		
Occupation	Government	49 (27.2%)	29 (38.2%)	1.00	–		–
	Unemployed	104 (57.8%)	27 (35.5%)	0.4 (0.2–0.7)	0.00	2.4 (0.1,5.8)	0.05*
	Self employed	27 (15.0%)	20 (26.3%)	0.4 (0.2–08)	0.05	2.7 (1.1–6.5)	0.03*
Monthly income (ETB)	< 600	90 (50.0%)	26 (34.2%)	0.5 (0.3–1.0)	0.06	1.0 (0.4,2.6)	0.9
	601–1500	44 (24.4%)	24 (31.6%)	1.00	–	1.2 (0.6,2.6)	0.6
	> 1501 birr	46 (25.6%)	26 (34.2%)	0.5 (0.3–0.8)	0.04	–	–
Types of Oil used	Palm oil	149 (82.8%)	61 (80.3%)	0.8 (0.4–1.7)	0.63		
	Cereal oil	31 (17.2%)	15 (19.7%)	1.00	–		
Work involving vigorous intensity of activity	Yes	78 (43.3%)	42 (55.3%)	1.00	–		
	No	102 (56.7%)	34 (44.7%)	0.6 (0.4–1.1)	0.08		
Regular physical exercise	Yes	57 (31.7%)	26 (34.2%)	1.00	–		
	No	123 (68.3%)	50 (65.8%)	0.9 (0.5–1.6)	0.7		
Family history of chronic disease	Yes	68 (37.8%)	21 (27.6%)	0.6 (0.3–1.1)	0.12		
	No	112 (62.2%)	55 (72.4%)	1.00	–		
Duration since Diabetic mellitus Diagnosed	< 1 Years	11 (6.1%)	11 (14.5%)	1.00	–	2.7 (1.1,7.1)	0.04*
	1–5 Years	99 (55.0%)	37 (48.7%)	0.9 (0.5–1.6)	0.81	–	–
	> 6 Years	70 (38.9%)	28 (36.8%)	0.4 (0.1–1.6)	0.03	1.1 (0.6,1.9)	0.8
Duration since anti Diabetic started	< 1 Year	13 (7.2%)	11 (14.5%)	1.00	–		
	1-5 Year	97 (53.9%)	38 (50.0%)	1.0 (0.6–1.8)	0.95		
	> 6 Years	70 (38.9%)	27 (35.5%)	0.5 (0.2–1.1)	0.09		
Total number of medications	1	76 (42.2%)	37 (48.7%)	1.00	–		
	2	70 (38.9%)	22 (28.9%)	0.5 (0.1–2.5)	0.4		
	3	26 (14.4%)	15 (19.7%)	0.8 (0.2–4.0)	0.8		
	4	8 (4.4%)	2 (2.6%)	0.4 (0.8–2.3)	0.3		

*P < 0.05

Based onWHO criteria female sex was (AOR = 0.4 at 95%CI: 0.2, 0.7, *P* = 0.000) significantly associated with MetS compared to male sex. Patients who were single were significantly associated with MetS and were about seventeen (AOR = 17 at 95%CI: 1.8, 166, *P* = 0.01) times more likely to develop MetS compared to those divorced patients.Details are presented in Table 5.

**Table 5:** Bivariable and multivariable logistic regression analysis result using WHO criteria at UGCSH, June 2017

Variable		Mets		COR 95%CI	*P* value	AOR 95%CI	*P*-value
		Yes	No				
Sex	Male	34 (29.3%)	79 (56.4%)	1.00	–		
	Female	82 (70.7%)	61 (43.6%)	0.3 (0.2–0.5)	0.00	0.4 (0.2,0.7)	< 0.000*
Age	< 30	10 (8.6%)	17 (12.1%)	1.00	–		
	31–40	6 (5.2%)	9 (6.4%)	0.5 (0.2–1.2)	0.12		
	41–50	23 (19.8%)	33 (23.6%)	0.6 (0.2–1.7)	0.31		
	51–64	41 (35.3%)	51 (36.4%)	0.6 (0.3–1.2)	0.13		
	> 65	36 (31.0%)	30 (21.4%)	0.7 (0.4–1.3)	0.21		
Residency	Urban	94 (81.0%)	113 (80.7%)	0.9 (0.5–1.8)	0.94		
	Rural	22 (19.0%)	27 (19.3%)	1.00	–		
Educational status	Unable to read and write	43 (37.1%)	33 (23.6%)	0.4 (0.2–0.7)	0.00	–	–
	Primary school	43 (37.1%)	44 (31.4%)	1.00	–	0.9 (0.5,1.9)	0.9
	Secondary school and above	30 (25.9%)	63 (45.3%)	0.8 (0.4–1.4)	0.4	1.7 (0.8,3.3)	0.2
Marital status	Single	7 (6.0%)	11 (7.9%)	0.06 (0.0–0.51)	0.01	17 (1.8166)	0.01*
	Married	92 (79.3%)	127 (90.7%)	0.5 (0.02–10.3)	0.7	14 (1.8113)	0.01*
	Divorced	6 (5.2%)	1 (0.7%)	1.00	–	–	–
	Widowed	11 (9.5%)	1 (0.7%)	0.05 (0.00–0.6)	0.01	2.0 (0.1,38.8)	0.6
Occupation	Government	31 (26.7%)	47 (33.6%)	1.00	–		
	Unemployed	68 (58.6%)	63 (45.0%)	0.5 (0.3–1.04)	0.06		
	Self employed	17 (14.7%)	30 (21.4%)	0.6 (0.3–1.1)	0.08		
Monthly income (ETB)	< 600 birr	56 (48.3%)	60 (42.9%)	1.00	–		
	601–1500 birr	29 (25.0%)	39 (27.9%)	1.2 (0.7,2.2)	0.5		
	> 1501 birr	31 (26.7%)	41 (29.3%)	0.9 (0.5,1.9)	0.9		
Types of Oil used	Palm oil	96 (82.8%)	114 (81.4%)	0.9 (0.5–1.7)	0.8		
	Cereal oil	20 (17.2%)	26 (18.6%)	1.00	–		
Work involving vigorous intensity of activity	Yes	49 (42.2%)	1 (50.7%)	1.00	–		
	No	67 (57.8%)	69 (49.3%)	0.7 (0.4–1.16)	0.2		
Regular physical exercise	Yes	36 (31.0%)	47 (33.6%)	1.00	–		
	No	80 (69.0%)	93 (66.4%)	0.9 (0.5–1.5)	0.66		
Family history of chronic disease	Yes	48 (41.4%)	41 (29.3%)	0.6 (0.3–0.9)	0.04	1.5 (0.9,2.6)	0.1
	No	68 (58.6%)	99 (70.7%)	1.00	–		
Duration since Diabetic mellitus diagnosed	< 1 Year	11 (9.5%)	11 (7.9%)	1.00	–		
	1-5 Years	63 (54.3%)	73 (52.1%)	0.9 (0.4–2.1)	0.74		
	> 6 Years	42 (36.2%)	56 (40.0%)	0.8 (0.3–1.9)	0.5		
Duration since anti Diabetic started	< 1 Year	12 (10.3%)	12 (8.6%)	1.00	–		
	1-5 Years	63 (54.3%)	72 (51.4%)	0.8 (0.5–1.4)	0.5		
	> 6 Years	41 (35.3%)	56 (40.0%)	1.1 (0.5–2.7)	0.8		
Total number of medications	1	45 (38.8%)	68 (48.6%)	1.00	–		
	2	47 (40.5%)	45 (32.1%)	0.9 (0.3–3.7)	0.99		
	3	20 (17.2%)	21 (15.0%)	1.6 (0.4–5.9)	0.50		
	4	4 (3.4%)	6 (4.3%)	1.4 (0.4–5.8)	0.61		

*P < 0.05

## Discussion

This study was aimed at describing the prevalence and predictors of Metabolic syndrome among type 2 diabetic patients attending a comprehensive specialized hospital in Northwest Ethiopia. The main finding of the present study demonstrates that MetS is a major health concern for diabetic patients in Ethiopia and the predictors like female gender, age between 51 and 64 years old, urban area residence, and being single, are modifiable.

The prevalence of MetS in this study was 70.3, 57 & 45.3% using NCEP-ATP III, IDF& WHO criteria respectively. These different prevalence rates arise due to the different cutoff points and sets of criteria used by those three definitions. In previous studies among DM patients, a lower 45.9% and comparable 70.1% results were reported from Ethiopia using NCEP-ATP III criteria [22, 23]. However, a higher rate of prevalence, 73.9, 69.9 and 66.8%, was reported from Nepal using NCEP-ATP III, WHO and IDF criteria respectively [30] and 73.4 & 64.9% using NCEP-ATP III and IDF criteria respectively was reported from Iran [31]. On the other hand, a lower prevalence of MetS was reported from India 45.8, 57.7 and 28% using NCEP-ATP III, WHO and IDF criteria respectively [8] and 58% was from Ghana using NCEP-ATP III criteria [7]. The prevalence of MetS in the present study some what different from others and this could be due to differences in sample size, socio-economic status, ethnicity difference [32], sampling method and difference in life style of study participants.

The present study demonstrated that prevalence of MetS was found to be higher in female (83.3, 66.1 & 70.7%) study participants than men (17, 31.9 & 29.3%) using IDF, NCEP-ATPIII & WHO criteria’s respectively. This result is in agreement with other studies [7, 30, 31]. As shown in Table 2, a significantly higher proportion of females than males have abnormal components in four (66.7%) of the six components used to define MetS in the three criterias. This might explain the observed higher prevalence of MetS in the female geneder. Such discrepancy is attributed to the several physiological differences: Pregnancy induced increase in weight as well as gestational DM; the use of hormonal oral contraceptives that can decrease insulin sensitivity, glucose tolerance, increase blood pressure and increase in weight gain; menopause promotes a change in body fat distribution to increase central adiposity [33]. However, as the majority of females in the present study (81.8%) were above 46 years old, the increased prevalence of MetS among females unlike that of males may be due to menopause. The presence of hormonal replacement therapy (HRT) was, however, not assessed but might have some effect on the higher prevalence of MetS. Inaddition, less proportion of females were involved in regular physical exercise than males in this study which might have its own contribution to the observed higher MetS prevalence among female. Females in Ethiopia are socio-economically and culturally influenced to stay at home so that they are typically involved in daily living activities rather than regular physical exercise to maintain body fitness. The role of exercise in minimizing risks of developing MetS is reported in Greec study of 1128 men and 1154 women [34].

According to the NCEP- ATPIII criteria, where the highest prevalence of MetS was observed, TG and HDL were the most frequent abnormal MetS components. Abnormal levels of TG and HDL has been implicated with adverse health effects. Fore example, Callaghan et al. reported that hypertriglyceridemia is a significant risk factor for lower-extremity amputation in a 10-year cohort study (from 1995 to 2006) of 28,701 diabetic patients [35]. A 2 years of multi-ethnic study of atherosclerosis on a total of 6814 participants showed that low level of HDL in the body is associated with an increased risk of CVD, coronary heart diseases and death [36]. Thus, interventions focusing on abnormal TG and HDL need to be prioritized.

Regarding to residency, the association of MetS and urbanization could be as a result of a sedentary life style, increased intake of calorie rich foods and central obesity.This result is supported by other studies world wide [37, 38]. In addition, people who were self employed had significant association with MetS and the reason could be also sedentary life style related with the type of job they are involved .

On the other hand, patients who were secondary school and above had significantly associated with MetS.This might be due to significantly higher economic status (greater than 1500 ETB) of those who are highly educated in our study population (Secondary school and above:59 (77.6%); primary school:15 (19.7%). This finding is consistent with that of Chakraborty et al. and Khanam et al. [39, 40]. Therefore, higher levl of education may indirectly lead to risky life style adoption interms of dietary pattern and physical activity.

When compared to those patients aged 30 years and less, the ones in the age intervals 31–40, 41–50, and 51–64 were at inceased risk of MetS. The reasons for a direct relations of age and MetS is that age related processes such as gradual decrease in the basal metabolic rate, stress induced hypercortisolism, hypogonadism, decreased growth hormone secretion, concomitant insulin resistance and abdominal fat deposition [41, 42]. However, those patients who are 65 years old and above were found to have no significantly increased risk of MetS. This might be because of reduced survival of patients who developed MetS in this age group. In this regard, further prospective studies need to be carried out. The finding reiterates that of Devers et al. According to this study which was conducted among 1429 adults aged ≥25 years from randomly selected house holds in Australia, MetS components cluster most markedly in those aged < 65 years [43]. Therefore, serious preventive and control measures should be taken as age increases. Individuals should be advised to make life style changes. Doing reguar exercise, eating foods containing little amount of saturated fats and cholesterol as well as taking more fiber-rich foods should be encouraged. Chandalia et al. have shown that taking high fiber diets have the potential to lower fasting plasma glucose, total cholesterol, triglyceride, and helps to have good glycemic index through a decrease in gastrointestinal absorption of cholesterol and carbohydrates [44].

Regarding to duration of period since DM was diagnosed, those patients diagnosed within a year had significantly higher risk of developing MetS according to NCEP-ATPIII criteria. Since lifestyle modifications on diet and physical activity are the main initial interventions in T2DM patients, those respondents treated for short period of time may not effectively adopt the needed life style changes and hence are at increaed risk of MetS. It is also worth to note that some of the patients in our study might be in the very ealy stages of treatment so that reduction of MetS components might be unlikely. Incontrast to our finding, a previous study in Ethiopia reported the absence of impact of duration of treatment on MetS development [22]. Since in this study patients were classified based on higher cut off treatment duration i.e. below or above 10 years, it might fail to signify the impact of duration on MetS. On the otherhand, patients who stayed on treatment for short duration were not specifically isolated and compared with others who stayed longer on therapy. In this study using WHO criteria it indicate that patients who were single had association with MetS,the possible reason may be small sample size of this segment of respondants (*N* = 18,7%).

In general, the findings of the present stydy taken together showed that MetS is a mjor burden among T2DM patients in Ethiopia. Early identification of MetS among T2DM patients is of great importance since MetS imply increased risk of morbidities such as CVD,decreased quality of life, increased health care cost, as well as mortality. Therefore, UGCSH has to strengthen appropriate and targeted prevention strategies such as encouraging people to adopt dietary modification and physical activity which are reported to reduce occurrence and progression of MetS [45]. Inaddition, there should be a more frequent screening of patients for MetS components prior to full blow development of MetS.

This study for the first time in Ethiopia, employed three defining criteria for MetS and was able to highlight the importance of having unified definition to diagnose and make clinical decisions in the context of low income settings. Data were also collected prospectively and this strengthens the conclusions made. Yet, there are limitations and one should consider these in interpreting the findings. The study may not be generalized to the nation as a whole due to small sample size and thus further studies would be important. It is also important to show the health related outcome and economic consequences of MetS among T2DM patients in Ethiopia.

## Conclusions

In conclusion, this study demonstrates that MetS is a major health concern for diabetic patients in Ethiopia. They are at increased risk of developing complications such as cardiovascular diseases and premature mortality.The predictors, female gender, age between 51 and 64 years old, urban area residence, and being single, are modifiable. Thus, health authorities shall provide targeted interventions to this most at risk sub populations of diabetic patients such as promotion of life style modifications.

## Abbreviations

CVD: Cardiovascular Diseases
DM: Diabetes Mellitus
MetS: Metabolic Syndrome
UGCSH: University of Gondar Comprehensive Specialized Hospital
